# Feasibility of a standardized family participation programme in the intensive care unit: A pilot survey study

**DOI:** 10.1002/nop2.1603

**Published:** 2023-01-08

**Authors:** Boukje Dijkstra, Lucia Uit het Broek, Johannes van der Hoeven, Lisette Schoonhoven, Frank Bosch, Marijke Van der Steen, Paul Rood, Lilian Vloet

**Affiliations:** ^1^ Research Department Emergency and Critical Care, HAN University of Applied Sciences School of Health Studies Nijmegen The Netherlands; ^2^ Department of Intensive Care Radboud University Medical Center Nijmegen The Netherlands; ^3^ Acute Care Unit Canisius‐Wilhelmina Hospital Nijmegen The Netherlands; ^4^ Nursing Science, Julius Center for Health Sciences and Primary Care University Medical Center Utrecht, Utrecht University Utrecht The Netherlands; ^5^ School of Health Sciences, Faculty of Environmental and Life Sciences University of Southampton Southampton UK; ^6^ Department of Intensive Care Rijnstate Arnhem The Netherlands; ^7^ Section Acute Internal Medicine, Department of Internal Medicine Radboud University Medical Center Nijmegen The Netherlands; ^8^ Department of Intensive Care Maasziekenhuis Pantein Boxmeer The Netherlands; ^9^ Department of Intensive Care Hospital Gelderse Vallei Ede The Netherlands; ^10^ IQ healthcare, Radboud Institute for Health Sciences Radboud University Medical Center Nijmegen The Netherlands; ^11^ Foundation Family and Patient Centered Intensive Care Alkmaar The Netherlands

**Keywords:** essential care, nursing, family‐centred care, family participation, implementation, intensive care unit, pilot study, relatives

## Abstract

**Aim:**

To assess the feasibility and applicability of a standardized programme to facilitate family participation in essential care activities in the intensive care unit.

**Design:**

Pilot study with a cross‐sectional survey design.

**Methods:**

A standardized programme to facilitate family participation in essential nursing care activities was implemented in intensive care units of three hospitals in the Netherlands from November 2018 until March 2019. The feasibility and applicability of the programme were assessed with surveys of the patients, relatives and healthcare providers.

**Results:**

Three intensive care units successfully implemented the standardized programme. Three patients, ten relatives and 37 healthcare providers responded to the surveys. Patients appreciated family participation and recognized that their relatives liked to participate. Relatives appreciated being able to do something for the patient (80%) and to participate in essential care activities (60%). The majority of relatives (60%) felt they had sufficient knowledge and skills to participate and did not feel obliged nor uncomfortable. Healthcare providers felt they were trained adequately and motivated to apply family participation; application was perceived as easy, clear and relatively effortless according to the majority. According to 68% of the healthcare providers, most relatives were perceived to be capable of learning to participate in essential care activities. Some healthcare providers felt uncertain about the patient's wishes regarding family participation, with some indicating the behaviours of relatives and patients discouraged them from offering family participation. Use of a standardized programme to facilitate family participation in essential care activities in the intensive care unit seems feasible and applicable as determined by relatives and healthcare providers.

## 1 INTRODUCTION

Admission to the intensive care unit (ICU) is frequently experienced as stressful and may negatively impact patients and their relatives. Many ICU survivors face long‐term consequences such as impairments of physical, cognitive and/or mental nature, addressed as “postintensive care syndrome” (PICS; Needham et al., 2012). Additionally, the majority of relatives report symptoms of anxiety, depression and/or posttraumatic stress related to the ICU stay, aggregated as PICS‐Family (PICS‐F), which may last for years (Davidson et al., 2012). Prevalence of mental health impairments among relatives varies from 13% to 56% (Davidson et al., 2012; Harvey & Davidson, 2016; Needham et al., 2012).

Stress among relatives of ICU patients should be addressed by healthcare providers (HCPs), taking their circumstances into account, with specific attention for mental strength. According to Zante et al. (2020), future research should be directed at individualized prevention of PICS‐F.

## 2 BACKGROUND

Postintensive care syndrome‐Family may be reduced by decreasing the relatives' anxiety and stress during the patient's ICU stay with help of family participation. ICU HCPs currently lack practical guidance to facilitate family participation in essential care activities in the ICU, and the effects of family participation interventions are unclear (Davidson et al., 2017). According to Olding et al. (2016), family involvement is a continuum, ranging from passive (“presence”) to active forms (“contribution to care”), where the latter corresponds to family participation in essential patient care activities. Potential essential care activities are, for example, application of lotion, combing hair or mobilization (Kitson et al., 2010).

Family participation in essential care may be considered a complex intervention, since it requires both ICU HCPs and relatives to change their behaviour. Furthermore, according to the MRC framework, it needs to be tailored to individual needs (Craig et al., 2008). A step in the development of this intervention is to determine the needs, perceptions, preferences and capacities of all involved, recently added to the MRC framework (Bleijenberg et al., 2018). This was recently done by our group Dijkstra et al. (2022). Based on the results of this review (focus group), interviews with former ICU patients, their relatives and ICU HCPs, a standardized programme to facilitate family participation in essential care activities in the ICU was developed.

This pilot study aimed to assess the feasibility and applicability of a standardized programme to facilitate family participation in essential care activities in the ICU.

## 3 METHOD

### 3.1 Study design

This was a pilot study with a cross‐sectional survey design to assess the feasibility and applicability of an intervention.

### 3.2 Setting and population

This study focused primarily on relatives and HCPs, of the ICUs of one university, one general teaching and one general hospital in the Netherlands from November 2018 until March 2019, since they play a major role in family participation. Relatives of adult ICU patients who had been in the ICU for more than one day, and if family participation was considered feasible, were invited to participate in essential patient care and asked to complete a survey after participating in an essential care activity. Relatives of patients that received palliative care were invited to participate; however, they were not asked to complete a survey for ethical reasons. ICU HCPs were asked to complete a survey when a relative had participated in an essential care activity. When possible, ICU patients were approached for a survey as well, if their mental and physical condition allowed this.

### 3.3 Standardized programme

A standardized programme to facilitate family participation in essential care activities in ICU was developed. The final programme contained 33 activities, including communication, amusement/distraction, comfort, personal care, breathing, mobilization and nutrition (Table 1). These items were based on existing literature (Kitson et al., 2010; Wyskiel, Weeks, et al., 2015), interviews with relatives and former ICU patients and focus groups with ICU HCPs. The programme was provided on a poster and complemented by information letters for relatives and ICU HCPs, underpinning safety precautions and the voluntary nature. Relatives could choose from the list of activities in consultation with the attending ICU nurse, physical therapist or speech therapist, taking the patient's situation at that moment into account. For example, when a patient was on high mechanical ventilation settings, relatives could apply body lotion, and when a patient was in a more stable condition, relatives were able to assist the patient with communication via a tablet, according to the ICU nurse's discretion.

**TABLE 1:** Menu for family participation in essential care activities

Communication	Helping with operating the tablet/ iPad
	Helping with the use of the letter board
	Helping with writing
	Being present during daily rounds^a^
Amusement/distraction	Reading from a book, newspaper or magazine
	Playing music or audiobook
	Switching on a favourite TV programme and watching it together
Comfort	Shaking the pillow(s)
	Applying body lotion on hands
	Applying body lotion on arms
	Applying body lotion on legs
	Giving a hand massage
	Giving a head massage
	Giving a foot massage
Care	Putting on glasses
	Putting in hearing aids
	Taking care of nails
	Helping taking care of lips
	Helping with mouth care
	Helping with dental care
	Helping with shaving
	Helping with combing hair
	Helping with washing hair
	Helping with washing
Breathing	Helping with breathing exercises
Mobilization	Helping with moving hands
	Helping with moving arms
	Helping with moving feet
	Helping with moving legs
	Helping with mobilization (changing position in bed)
	Helping with mobilization (on the bed edge)
	Helping with mobilization (in the chair)
	Helping with mobilization (walking)
	Helping with mobilization (in the swimming pool)^a^
Nutrition	Being present at and helping with eating and drinking

^a^ Available in one setting only.

### 3.4 Implementation strategy

Each hospital assembled a local project team for implementation of the programme, consisting of ICU nurses, physicians and/or physical therapists. These teams received a training and working method from the researcher and could tailor prefabricated posters, (digital) brochures and the implementation strategy to local wishes. Relatives received written and verbal instructions on participation in essential care activities from the local project team members and local ICU nurses. Relatives' preferences and arrangements regarding family participation were recorded on the poster and/or in the patient's file and assessed regularly.

### 3.5 Data collection

Data were collected through surveys. Surveys were developed for patients, relatives and HCPs separately, based on the *comprehensive, integrated checklist of determinants of practice* (TICD checklist) (Flottorp et al., 2013; Wensing & Grol, 2017), and focused on intervention and activities, opinions of patients, relatives and ICU HCPs, the information provided for relatives, experiences in practice, changes in care and contextual factors.

The survey for patients consisted of five items of which four TICD items were multiple‐choice questions with the possibility of adding free text. The fifth question asked patients to share whatever they wanted with regard to family participation. The number of items was deliberately kept low, to minimize burden, as most former ICU patients suffer from limited physical, cognitive and emotional capacities.

The survey for relatives contained 36 items in four major areas: intervention and activities, opinions of relatives and patients, the information provided for relatives and the provided instrument and experiences with family participation in practice.

The survey for ICU HCPs contained 56 items in four major areas: interventions and activities, opinions of professionals, patients and relatives, changes in care and contextual factors. The survey consisted of multiple‐choice questions with the possibility of adding free text and open questions.

The surveys were pilot tested by a group of experts, with expertise in (critical care) nursing, critical care medicine, survey development and implementation research to establish face validity. Some minor adjustments were deemed necessary, implying face validity. As this was a pilot study, reliability was not established; however, for optimal reliability, the questions were based on the checklist for identifying determinants of practice, developed by Flottorp et al. (2013).

Surveys were distributed to the participating hospitals 2 weeks after start of the study. The local project team members asked relatives and ICU HCPs to complete surveys during ICU stay, after each moment of participating. The survey was handed to patients only once, on the day of ICU discharge, when patients were able to answer the questions in the survey, possibly with assistance of a relative or ICU nurse. Completion of a survey was considered consent.

### 3.6 Data analysis

Quantitative data of the surveys were analysed using SPSS (version 25) and presented using descriptive statistics. Qualitative data were thematically coded based on the TICD checklist (Flottorp et al., 2013). Statistics and themes resulting from the surveys were discussed and explored further in the stakeholders meeting.

### 3.7 Ethical considerations

The study was approved by the Research Ethics Committee (REDACTED) and subsequently by the Hospital Ethics Committees of the participating ICUs.

## 4 RESULTS

A total of three patients, ten relatives and 37 ICU HCPs completed the surveys within the planned period. Both relatives and ICU HCPs were mostly female. Most relatives were spouses (80%). ICU HCPs were predominantly ICU nurses (78%) and had six or more years of working experience (64%; Table S1).

### 4.1 Patients

Patients liked family participation and recognized their relative liked to participate. Patients were not worried that participating in care was too stressful for their relative and experienced that ICU nurses had sufficient time to facilitate family participation.

### 4.2 Relatives

Relatives participated in nearly all possible care activities, except for washing the patient's hair.

Relatives liked being able to do something for the patient (80%) and to participate in essential care activities (60%). More than half felt invited by the ICU nurse or other ICU HCPs to participate. Some relatives indicated that participating in care helped them to feel involved and that it felt familiar for both patient and relative. The majority (60%) felt they had sufficient knowledge and skills to participate and did not feel obliged nor uncomfortable (Table S2).

### 4.3 Professionals

Most ICU HCPs had a neutral or positive attitude towards family participation in essential care activities (81%).

Most felt they were trained adequately (92%), had sufficient knowledge and skills (67%–78%) and felt motivated to apply family participation (76%). Application was perceived to be easy, clear and relatively effortless according to the majority (62%–76%).

Reading aloud, applying body lotion and presence/assistance with eating and drinking were applied most frequently.

Uncertainty about the patient's wishes regarding family participation played a role for nine ICU HCPs (24%). According to ICU HCPs (68%), most relatives were perceived to be capable of learning to participate in essential care activities, 46% felt that some activities may be unsafe in some situations and require professional support, none reported harms. Some ICU HCPs (16%) indicated that behaviour of relatives and/or patients discouraged them to offer family participation (Table S3).

## 5 DISCUSSION

A programme to facilitate family participation in essential care activities in the ICU is feasible and applicable in daily ICU practice, according to relatives and ICU HCPs.

Patients involved in this study had a positive attitude towards family participation, similar to the findings of Garrouste‐Orgeas et al. (2010), though only few patients were able to answer the questions in our short survey. Knowledge about patients' needs, perceptions and preferences, with regard to family participation in essential care, remains limited (Dijkstra et al., 2022; Olding et al., 2016). Most patients have an altered state of consciousness either due to sedatives or the underlying condition, limiting them to express needs, perceptions and preferences. While challenging, enhanced insights into the patient's perspective herein may further optimize their rehabilitation in the future.

The majority of relatives appreciated being able to help the patient and participate in care activities. The results in this study correspond with findings from previous studies (Azoulay et al., 2003; Blom et al., 2013; Eldredge, 2004; Garrouste‐Orgeas et al., 2010; Hammond, 1995; Hupcey, 1999; Mitchell & Chaboyer, 2010; Wong et al., 2019, 2020; Wyskiel, Chang, et al., 2015). These results imply that providing the opportunity to participate in care activities satisfies the needs of relatives. Knowledge on possible effects of family participation on relatives is still scarce though. Amass et al. (2020) and Skoog et al. (2016) found promising results in their studies, where family participation in essential care activities was associated with a significant reduction in mental health symptoms. Use of other outcome measures may be considered as well, since family participation may affect relatives in other ways (Dijkstra et al., 2023 under review).

A majority, 76%–81%, of ICU HCP had a neutral to positive attitude towards family participation (81%) and was motivated to apply it in daily practice (76%), as established in previous studies (Azoulay et al., 2003; Garrouste‐Orgeas et al., 2010; Hammond, 1995; Hetland et al., 2017; Kean & Mitchell, 2014; Wyskiel, Chang, et al., 2015). Several intervention studies assessed additional outcomes from the ICU HCPs' perspective. According to Wyskiel, Chang, et al. (2015), most ICU HCPs considered various care activities appropriate, though only few invited relatives to participate all the time. Mitchell et al. (2017) found that ICU nurses favoured family participation in general; ICU nurses in the study of Amass et al. (2020) thought that the intervention improved their communication with relatives and did not interfere with patient care.

The response rate among relatives in our study was relatively low. Davidson et al. (2010) and Mitchell et al. (2017) encountered similar issues assessing feasibility in their studies, with fear and discomfort among relatives and difficulties in recruitment. Since an ICU stay is stressful for relatives, recruitment will remain challenging and requires appropriate timing in an unpredictable setting.

A stakeholder's meeting with members of the project teams and other ICU HCPs took place at the end of the implementation phase. During this meeting, the survey results were assessed. From this stakeholder's meeting, a general theme emerged that applying family participation is dependent on the situation, and the patient's, relatives' and ICU HCP's circumstances should be taken into account. Minor adjustments were made in the working method, to address safety concerns and enhance support.

A strength of this study is that it was conducted in three centres that differed in several aspects, such as the number of patients per ICU, visiting hours and single bedrooms versus multiple bedrooms. This variability assures that the developed programme is feasible and applicable in various ICU settings. Another strength was the implementation strategy, which was structured according to the TICD checklist (Flottorp et al., 2013), but allowed for local tailoring, which enhanced application through earlier successful local pathways.

Also, some limitations have to be addressed. First, since patients and their relatives were included through purposive sampling and willingness to participate and complete the survey, this may have led to selection bias. However, we tried to include a wide variety of patients and relatives while minimizing their burden, which is probably an adequate reflection of the potential future target population, reducing bias as much as reasonably possible (Polit & Beck, 2020; Portney & Watkins, 2013). Second, the number of responses collected was limited. However, we feel that we obtained sufficient insight in the programme's feasibility and applicability. For insights into the effects of the programme, a larger scale trial is warranted. Third, project team members were mostly part of the HCP team in the ICU, which may have resulted in role conflicts. However, this effect is probably bidirectional, as implementing and adapting the programme to local standards may have enhanced local adoption.

## 6 CONCLUSIONS

Use of a standardized programme to facilitate family participation in essential care activities in the ICU seems feasible and applicable as determined by relatives and ICU HCPs. Several barriers and facilitators should be considered when the programme is applied, requiring tailoring to the individual needs and wishes of patients and relatives. Furthermore, possibilities for family participation require assessment of the situation at that time, regarding the patient and ward, allowing ICU HCPs to provide sufficient time and support.

## AUTHOR CONTRIBUTIONS

B.D., L.B. and L.V. contributed to the conception and design of the study. B.D., L.B., F.B., M.S. and L.V. contributed to the collection of data. B.D., L.B., P.R. and L.V. contributed to the data analysis. All authors contributed to the interpretation of the data and to the development of the first draft of the article. Subsequent drafts were reviewed and approved by all authors.

## FUNDING INFORMATION

This project was funded by the Dutch Research Council (NWO) (RAAK.PUB03.011).

## CONFLICT OF INTEREST

No conflict of interest has been declared by the authors.

## PATIENT OR PUBLIC CONTRIBUTION

Patients and relatives participated in the conduct of the study.

## Supporting information


Table S1‐S3

## Data Availability

The datasets used and analysed during the current study are available from the corresponding author on reasonable request.
