# Supplementary material for: Feasibility of a standardized family participation programme in the intensive care unit: A pilot survey study
Source: Nurs Open. 2023 Jan 8;10(6):3596–602. doi: 10.1002/nop2.1603 (PMC10170932; doi:10.1002/nop2.1603)
Supplement: Supplementary file 1 — Table S1‐S3 [file NOP2-10-3596-s001.docx]

**Supplemental Table 1 Demographic data relatives and healthcare professionals**

|  | **Relatives**  (n=10) | **Professionals**  (n=37) |
| --- | --- | --- |
| **Sex** |  |  |
| Female | 7 (70%) | 30 (81%) |
| Missing | 1 (10%) |  |
| **Relationship** |  |  |
| Spouse | 8 (80%) |  |
| Child | 2 (20%) |  |
| **Age (in years)** |  |  |
| 35-44 | 2 (20%) |  |
| 45-54 | 1 (10%) |  |
| 55-64 | 1 (10%) |  |
| 65-74 | 3 (30%) |  |
| >75 | 1 (10%) |  |
| **Healthcare profession** |  |  |
| ICU nurse |  | 29 (78%) |
| ICU nurse in training |  | 2 (5%) |
| Physiotherapist |  | 4 (11%) |
| Nurse |  | 1 (3%) |
| Missing |  | 1 (3%) |
| **Years of working experience** |  |  |
| <1 year |  | 5 (14%) |
| 1-5 year |  | 8 (22%) |
| 6-10 years |  | 9 (24%) |
| >10 years |  | 15 (40%) |
| **Educational level*** |  |  |
| Vocational | 2 (20%) | 5 (14%) |
| Bachelor | 3 (30%) | 13 (35%) |
| Post-bachelor/certification | 2 (20%) | 16 (43%) |
| Different | 1 (10%) | 1 (3%) |
| Missing | 2 (20%) | 2 (5%) |
| **Emergency ICU admission** |  |  |
| Yes | 10 (100%) |  |
| **Hospital** |  |  |
| University |  | 3 (8%) |
| General |  | 33 (89%) |
| Missing |  | 1 (3%) |

**Supplemental Table 2 Results survey relatives**

| Statement | Yes/Agree | Disagree |
| --- | --- | --- |
| Opinions of relatives (n = 9) |  |  |
| I would like to help in essential patient care for my significant other | 6 (67%) |  |
| I appreciated being able to do something in the care for my significant other | 8 (89%) |  |
| I felt free in my choice to help in essential patient care | 8 (89%) |  |
| My relative appreciated that I could do something for him/her in essential patient care | 8 (89%) |  |
| Did you feel invited by the ICU nurse to help in essential patient care? | 6 (67%) |  |
| Did you feel invited by other healthcare providers to help in essential patient care? | 5 (56%) |  |
|  |  |  |
| The information provided for relatives and the provided tool (n = 8) |  |  |
| The lay-out of ‘information for relatives’ is clear | 6 (75%) | 1 (13%) |
| The way ‘information for relatives’ was offered is practical/usable | 7 (87%) |  |
| The ‘information for relatives’ provided with me with sufficient clarity about the possibilities to help in essential patient care | 8 (100%) |  |
| The lay-out of the ‘instrument’ is clear | 4 (50%) | 1 (13%) |
| The way in which the ‘instrument’ was offered is practical/usable | 6 (75%) |  |
| The ‘instrument’ provided with me with sufficient clarity about the possibilities to help in essential patient care | 6 (75%) |  |
|  |  |  |
| Experiences with family participation in practice (n = 8) |  |  |
| It was clear to me how I could help in essential patient care | 6 (75%) |  |
| Helping in essential patient care took me a lot of effort | 0 (0%) |  |
| I had enough knowledge to help in essential patient care | 6 (75%) |  |
| I had enough skills to help in essential patient care | 6 (75%) |  |
| The ICU nurse asked if I wanted to do something | 3 (38%) |  |
| I indicated that I wanted to do an activity from the ‘menu’ | 4 (50%) |  |
| I chose the activity of the ‘menu’ myself | 5 (63%) |  |
| I could do the activity that I wanted | 6 (75%) |  |
| The nurse encouraged me to help in ICU patient care for my significant other | 3 (38%) |  |
| I found it scary to help in ICU patient care for my significant other | 2 (25%) |  |
| I appreciated helping in ICU patient care for my significant other | 7 (87%) |  |
| I felt obliged to help in ICU patient care for my significant other | 2 (25%) |  |
| I felt that I could choose if I wanted to help | 8 (100%) |  |
| There was enough room in the ICU to help in ICU patient care for my significant other | 8 (100%) |  |
| There was enough privacy in the ICU to help in ICU patient care for my significant other | 8 (100%) |  |
| The visiting hours allowed me to help in basic care for my significant other | 6 (75%) |  |
| The ICU nurse had time to guide me through helping in ICU patient care for my significant other | 3 (38%) |  |

**Supplemental Table 3 Results survey ICU healthcare providers**

| Question/statement |  |  |  |  | Yes  (n (%)) | Missing (n (%)) |
| --- | --- | --- | --- | --- | --- | --- |
| Opinions of professionals, patients and relatives |  |  |  |  |  |  |
| Did you find it difficult to invite relatives to participation in essential patient care? |  |  |  |  | 3 (8%) | 5 (14%) |
| Did uncertainty about the wishes of the patient play a role in family participation in essential patient care (protection of privacy)? |  |  |  |  | 9 (24%) | 4 (11%) |
| Did uncertainty about relatives abilities play a role in family participation in essential patient care? |  |  |  |  | 9 (24%) | 6 (16%) |
| Was the relative capable of learning to participate in essential patient care? |  |  |  |  | 25 (68%) | 6 (16%) |
| Have you experienced behaviour of relatives that encouraged you to offer family participation in essential patient care? |  |  |  |  | 22 (60%) | 5 (14%) |
| Have you experience behaviour of relatives that discouraged you to offer family participation in essential patient care? |  |  |  |  | 6 (16%) | 4 (11%) |
| Have you experienced behaviour of the ICU patient that encouraged you to offer family participation in essential patient care? |  |  |  |  | 13 (35%) | 6 (16%) |
| Have you experience behaviour of the ICU patient that discourages you to offer family participation in essential patient care? |  |  |  |  | 6 (16%) | 3 (8%) |
| Were there activities you did not find safe for relatives to participate in? |  |  |  |  | 17 (46%) | 5 (14%) |
| Has your opinion about family participation changed? |  |  |  |  | 2 (5%) | 4 (11%) |
|  |  |  |  |  |  |  |
| Changes in care | **Strongly disagree** | **Disagree** | **Neutral** | **Agree** | **Strongly agree** | **Missing** |
| Family participation in essential patient care was easy to apply |  | 7 (19%) | 1 (3%) | 19 (51%) | 4 (11%) | 6 (16%) |
| It was clear how I could apply family participation in essential patient care |  | 3 (8%) | 1 (3%) | 18 (49%) | 9 (24%) | 6 (16%) |
| It was clear to relative how they could participate in essential patient care |  | 4 (11%) | 1 (3%) | 23 (62%) | 5 (14%) | 4 (11%) |
| The relative was satisfied with the participation in essential patient care |  |  | 3 (8%) | 22 (60%) | 7 (19%) | 5 (14%) |
| The patient was satisfied with the participation in essential patient care |  |  | 2 (5%) | 19 (52%) | 6 (16%) | 10 (27%) |
| I was satisfied with the participation in essential patient care of the relative |  | 1 (3%) | 1 (3%) | 21 (57%) | 8 (22%) | 6 (16%) |
| Family participation in essential patient care took a lot of effort | 7 (19%) | 13 (35%) | 3 (8%) | 8 (22%) |  | 6 (16%) |
| I had sufficient knowledge to apply family participation in essential patient care |  | 1 (3%) | 1 (3%) | 19 (51%) | 10 (27%) | 6 (16%) |
| I had sufficient skills to apply family participation in essential patient care |  | 3 (8%) | 3 (8%) | 16 (43%) | 9 (24%) | 6 (16%) |
| The relative had sufficient knowledge to apply family participation in essential patient care |  | 5 (14%) | 2 (5%) | 21 (57%) | 3 (8%) | 6 (16%) |
| The relative had sufficient skills to apply family participation in essential patient care |  | 7 (19%) | 4 (11%) | 16 (43%) | 4 (11%) | 6 (16%) |
| I was motivated to apply family participation in essential patient care |  | 1 (3%) | 2 (5%) | 17 (46%) | 11 (30%) | 6 (16%) |
| There were emotions that hindered the application family participation in essential patient care | 5 (14%) | 15 (41%) | 1 (3%) | 9 (24%) | 1 (3%) | 6 (16%) |
| Applying family participation in essential patient care took me extra time | 2 (5%) | 10 (27%) | 2 (5%) | 17 (46%) |  | 6 (16%) |
| My job satisfaction has increased through the application of family participation in essential patient care | 4 (11%) | 8 (22%) | 9 (24%) | 8 (22%) | 3 (8%) | 5 (14%) |
| The application of family participation in essential patient care fits within the current policy of the department | 1 (3%) | 4 (11%) | 2 (5%) | 21 (57%) | 5 (14%) | 4 (11%) |
| The application of family participation in essential patient care was a major change in my daily work | 2 (5%) | 25 (68%) | 2 (5%) | 4 (11%) | 1 (3%) | 3 (8%) |
| The team had sufficient skills to apply family participation in essential patient care | 1 (3%) | 3 (8%) | 2 (5%) | 26 (70%) | 1 (3%) | 4 (11%) |
| Family participation in essential patient care can save time in the long term | 5 (14%) | 15 (41%) | 2 (5%) | 13 (35%) |  | 2 (5%) |
| The quality of care improves through the application of family participation in essential patient care | 3 (8%) | 7 (19%) | 7 (19%) | 13 (35%) | 5 (14%) | 2 (5%) |
|  |  |  |  |  |  |  |
| Contextual factors | **Strongly disagree** | **Disagree** | **Neutral** | **Agree** | **Strongly agree** | **Missing** |
| There is enough room in the ICU to apply family participation in essential patient care | 1 (3%) | 4 (11%) | 1 (3%) | 25 (68%) | 4 (11%) | 2 (5%) |
| There is enough privacy in the ICU to apply family participation in essential patient care | 1 (3%) | 9 (24%) | 1 (3%) | 19 (51%) | 5 (14%) | 2 (5%) |
| Visiting hours facilitate the application of family participation in essential patient care | 7 (19%) |  |  | 22 (60%) | 5 (14%) | 3 (8%) |
| I had enough time during this shift to apply family participation in essential patient care |  | 9 (24%) |  | 20 (54%) | 5 (14%) | 3 (8%) |
| I feel supported by the members of the project team to apply family participation in essential patient care | 1 (3%) | 4 (11%) | 1 (3%) | 22 (60%) | 7 (19%) | 2 (5%) |
| I feel supported by my supervisor to apply family participation in essential patient care | 1 (3%) | 13 (35%) | 2 (5%) | 15 (41%) | 2 (5%) | 4 (11%) |
| The application of family participation in essential patient care fits within the values ​​of quality of care for patients in the ICU | 1 (3%) | 3 (8%) | 3 (8%) | 22 (60%) | 6 (16%) | 2 (5%) |
| The layout of the ‘instrument’ is clear | 1 (3%) | 4 (11%) |  | 21 (57%) | 6 (16%) | 5 (14%) |
| The way the ‘instrument’ is offered is practical / usable | 1 (3%) | 5 (14%) |  | 19 (51%) | 7 (19%) | 5 (14%) |
| The ‘instrument’ gives me sufficient guidance to apply family participation in essential patient care | 1 (3%) | 4 (11%) | 1 (3%) | 23 (62%) | 3 (8%) | 5 (14%) |
